# Survey of renewable chemicals produced from lignocellulosic biomass during ionic liquid pretreatment

**DOI:** 10.1186/1754-6834-6-14

**Published:** 2013-01-28

**Authors:** Patanjali Varanasi, Priyanka Singh, Manfred Auer, Paul D Adams, Blake A Simmons, Seema Singh

**Affiliations:** 1 Joint Bioenergy Institute, Physical Biosciences Division, Lawrence Berkeley National Laboratory, Emeryville, CA, USA; 2 Sandia National Laboratories, Biological and Materials Science Center, Livermore, CA, USA

**Keywords:** Lignin valorization, Ionic liquid pretreatment, Renewable chemicals, Biofuels

## Abstract

**Background:**

Lignin is often overlooked in the valorization of lignocellulosic biomass, but lignin-based materials and chemicals represent potential value-added products for biorefineries that could significantly improve the economics of a biorefinery. Fluctuating crude oil prices and changing fuel specifications are some of the driving factors to develop new technologies that could be used to convert polymeric lignin into low molecular weight lignin and or monomeric aromatic feedstocks to assist in the displacement of the current products associated with the conversion of a whole barrel of oil. We present an approach to produce these chemicals based on the selective breakdown of lignin during ionic liquid pretreatment.

**Results:**

The lignin breakdown products generated are found to be dependent on the starting biomass, and significant levels were generated on dissolution at 160°C for 6 hrs. Guaiacol was produced on dissolution of biomass and technical lignins. Vanillin was produced on dissolution of kraft lignin and eucalytpus. Syringol and allyl guaiacol were the major products observed on dissolution of switchgrass and pine, respectively, whereas syringol and allyl syringol were obtained by dissolution of eucalyptus. Furthermore, it was observed that different lignin-derived products could be generated by tuning the process conditions.

**Conclusions:**

We have developed an ionic liquid based process that depolymerizes lignin and converts the low molecular weight lignin fractions into a variety of renewable chemicals from biomass. The generated chemicals (phenols, guaiacols, syringols, eugenol, catechols), their oxidized products (vanillin, vanillic acid, syringaldehyde) and their easily derivatized hydrocarbons (benzene, toluene, xylene, styrene, biphenyls and cyclohexane) already have relatively high market value as commodity and specialty chemicals, green building materials, nylons, and resins.

## Background

**Figure 1:**
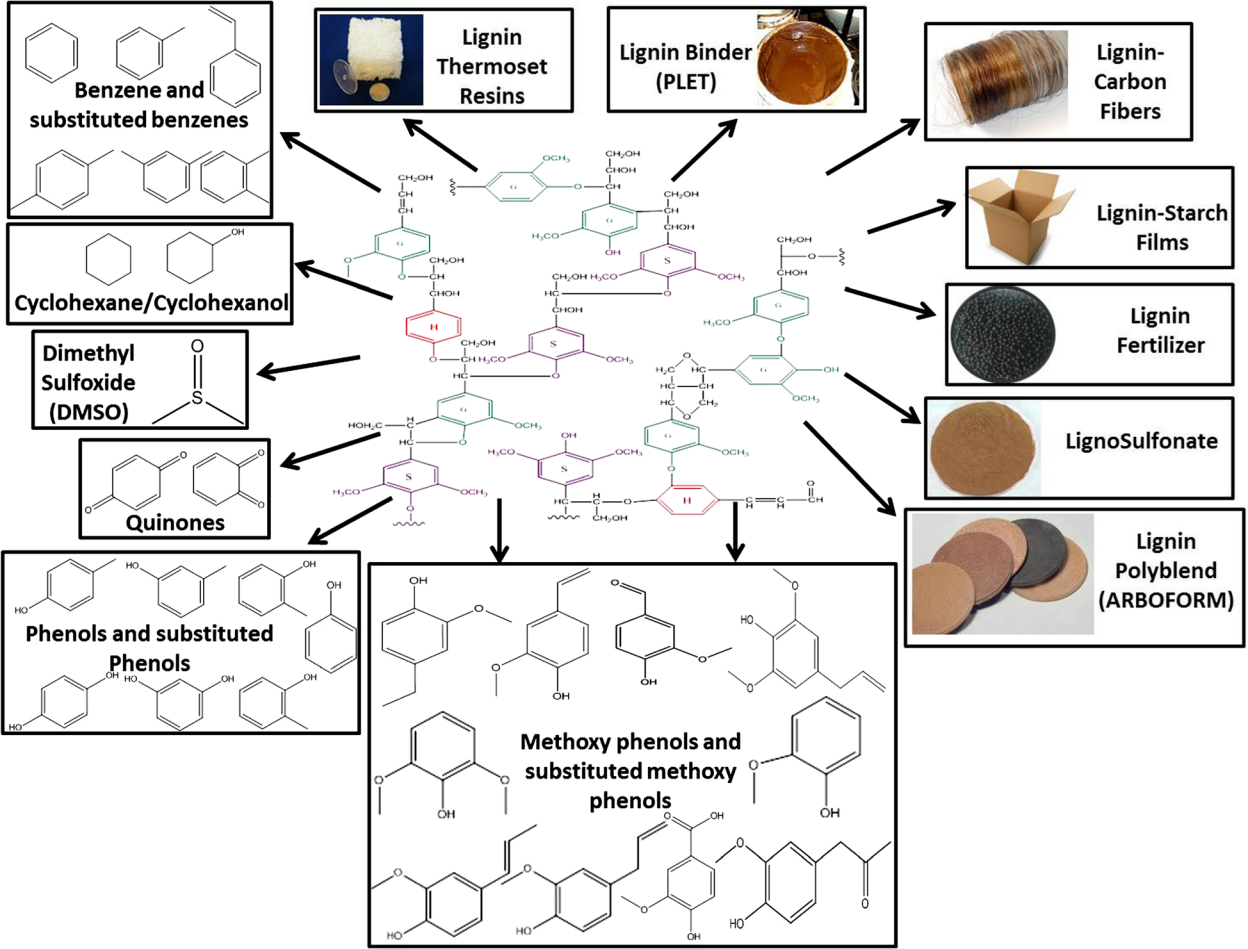
Schematic depiction of the different routes to convert lignin into renewable materials and chemicals.

Lignocellulosic biomass is primarily composed of three biopolymers: cellulose, hemicelluloses and lignin [1]. Holocellulosic biopolymers are considered the most valuable components of lignocellulose and are utilized for the production of various products including paper and biofuels [2-4]. Lignin constitutes roughly a third of the biomass and is typically burned to produce waste heat and/or electricity within paper mills and biorefineries [1,5]. Lignin is a naturally occurring heterogeneous phenylpropanoid-based biopolymer, and provides mechanical support and water transport to the plant and inhibits the action of various biological agents (e.g., insects) on the plants [6]. It is estimated that 50 million tons of lignin is produced annually from pulp and paper industries worldwide [7]. The high energy content of lignin, the presence of highly reactive groups, and the fact that it will be generated in large quantities as second generation biorefineries are deployed represents a significant opportunity for the production of a wide range of renewable chemicals and materials that can be sold as co-products (Figure 1).

For example, lignin sulfonates produced from kraft pulping are currently utilized as phenol-formaldehyde plastics, binders, adhesives, mud-sand cements in drilling oil-wells, dispersants, or flotation agents, emulsifiers and stabilizers, grinding agents, electrolytic refining agents, protein precipitants, tanning agents, sequestering agents, storage battery plates, lime plaster, crystal growth inhibitor, ingot mold wash and as flame retardants [8,9]. Starch-based films incorporated with lignin have higher water resistance and increased elongation that make them effective packaging materials [10]. Lignin is also used in the production of conducting polymer lignosulfonic acid-doped polyamine [11]. Carbon fibers produced from lignin based materials require a lower amount of thermo-stabilization and possess high tensile strength [12]. Lignin has been used to produce various polymers like ARBOFORM, polyesters and polyurethanes and various polymer blends with PVC, polyolefins, and rubbers are being currently developed [13-15]. Lignin has also been used as slow release nitrogenous fertilizers for soil and catalyst for the Kraft pulping process [10,16]. Due to its hydrophobic nature, lignin can be used in the manufacture of gypsum wallboards [10].

**Figure 2:**
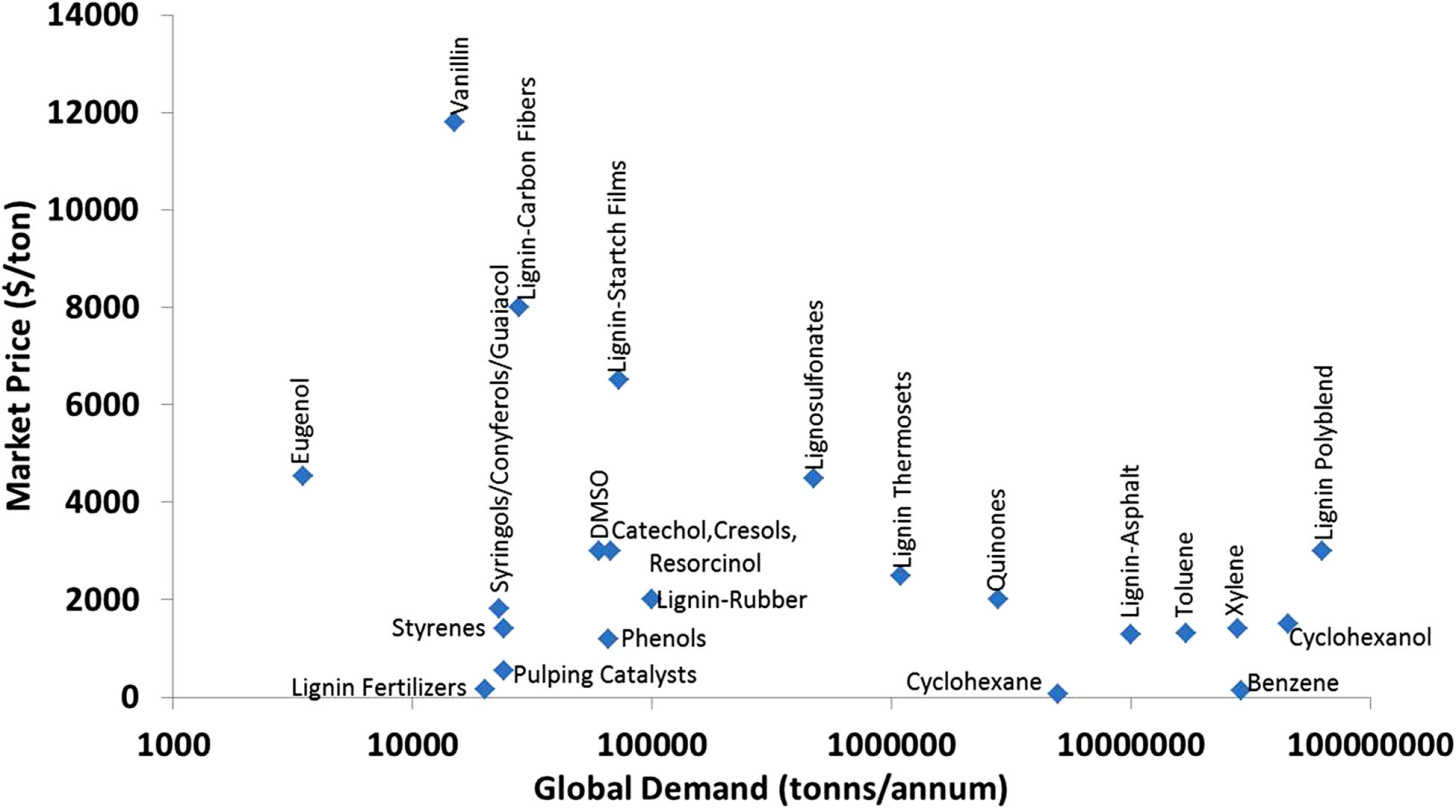
**Market price vs. demand for lignin-derived products**[24]**.**

In addition to these applications, lignin is a potential renewable source for many low molecular weight chemicals like benzene, phenol, guaiacol, vanillic acid, methanol, acetic acid, and dimethyl sulfoxide (DMSO) [17,18]. These lignin products are considered “value-added” chemicals (Figure 2) that could substantially impact the profit margins of a lignocellulosic biorefinery, but significant hurdles remain before they can be fully realized. One of the most significant of these is the realization of an efficient, cost-effective, and scalable means of fractionating lignocellulose into polysaccharide- and lignin-rich streams that enable downstream conversion into fuels and chemicals. Recently, some ionic liquids (ILs) have been shown to be very effective in the delignification of lignocellulosic biomass and depolymerization of lignin [19-23]. While much of the focus on ILs in the biomass field has been on their impact on the crystalline nature of cellulose, little attention has been paid in their potential utility as a means to valorize lignin.

Recent efforts in converting lignin to its monomeric products using ILs have focused on the technical lignins extracted from lignocellulosic biomass [25-27]. Cox and Ekerdt have shown that during IL dissolution, lignin depolymerization occurs through breakdown of alkyl-aryl ether linkages [26]. But no lignin breakdown products were reported to be observed during this process. Through electro-catalytic oxidative cleavage of lignin, Reichert et al. produced various aromatic compounds like guaiacol, vanilic acid, vanillin, acetovanillone, syringol, syringaldehyde, and syringic acid from alkali lignin [25]. Though a total yield of 6% was observed, no information about the relative quantities of each compound was reported. Stark et al. produce syringaldehyde and 2,6-dimethoxy-1,4-benzoquinone from oxidative depolymerization of Beech lignin. Although an impressive yield (66.3%) of the products was obtained the process utilized high pressure (84 × 10^5^ Pa of air) and very long reaction times (24 h) [27]. Lignin cleavage to monomers has also been accomplished using *Bacillus sp*. LD003 by Bandounas et al., but the incubation times for microbial degradation were very long (1–2 days) [28]. In this work, we investigate the ability of the IL 1-ethyl-3-methylimidazolium acetate ([C_2_mim][OAc]) to depolymerize lignin and produce valuable products from a series of technical lignins (kraft lignin, low sulfonate alkali lignin) as well as samples of switchgrass, pine and eucalyptus during pretreatment.

## Results and discussion

### Extraction of lignin byproducts from IL pretreatment using [C_2_mim][OAc]

Measured amounts of three different biomass types representing grasses, softwood and hardwood (switchgrass, pine, and eucalyptus respectively) and technical lignins (kraft and low sulfonate alkali) were treated with [C_2_mim][OAc] at 160 and 120°C for 6 hrs. Since IL and water are both polar, recovery of polar lignin breakdown products posed significant challenges. Our choice of extraction solvents for non-polar products included pentane, hexane, heptane and benzene. Out of the solvents tried, benzene enabled the most recovery and hence we have included the data on benzene extracted products in this report. Although yields were not the same using different solvents, patterns of lignin degradation and recovery were similar for all the solvents tested. Table 1 shows the percent biomass recovered for various types of biomass pretreated at 160°C. For all the conditions studied there is a loss of mass observed, indicating that lignin and other biomass constituents remain solubilized in the supernatant. At low biomass loading levels, low sulfonate alkali lignin showed the maximum solubilization, followed with switchgrass. Percent recoveries were found to be similar for kraft lignin and eucalyptus. Interestingly, at higher loadings, the extent of solubilization was found to be very different and the observed extent of solubilization at 20% loadings were switchgrass = eucalyptus > pine dust = low sulfonate alkali > kraft lignin. In addition, similar extent of mass solubilization from technical lignin and lignocellulosic biomass indicates high levels of impurities (other material than lignin) present in the technical lignins we used.

**Table 1:** Percent biomass recovered as a function of biomass loading during dissolution

**Biomass**	**Biomass loading during dissolution**	**% Recovered after dissolution**
**Kraft Lignin**	3%	60 ± 2
	10%	69 ± 7
	20%	84 ± 4
**Low Sulfonate Alkali Lignin**	3%	33 ± 1
	10%	43 ± 1
	20%	45 ± 3
**Switchgrass**	Untreated	N/A
	3%	40 ± 1
	10%	41 ± 4
	20%	35 ± 1
**Pine Dust**	Untreated	N/A
	3%	78 ± 5
	10%	56 ± 1
	20%	44 ± 5
**Eucalyptus**	Untreated	N/A
	3%	64 ± 1
	10%	58 ± 1
	20%	36 ± 1

### Lignin breakdown products from technical lignins

**Figure 3:**
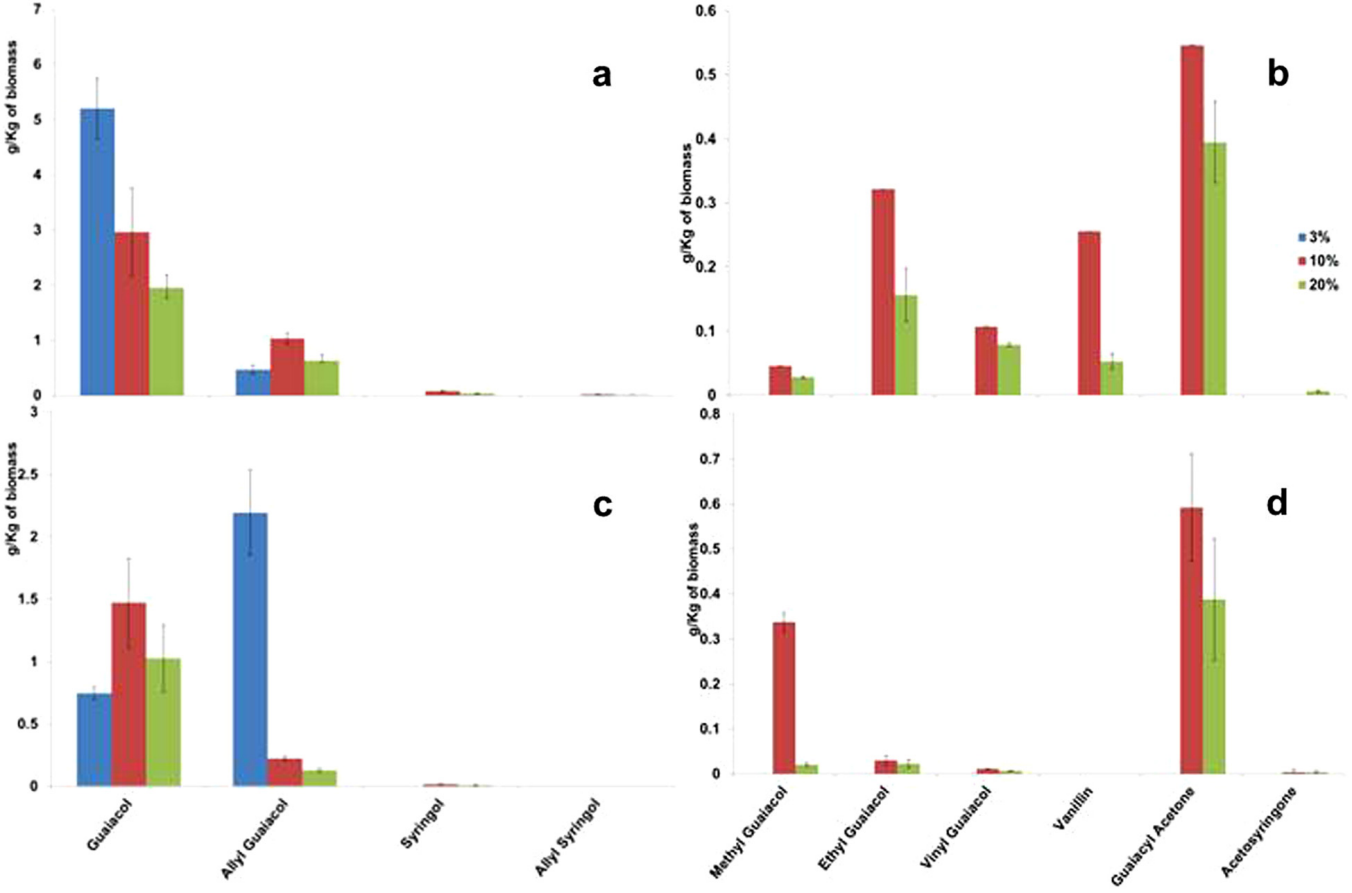
Major and minor lignin breakdown products from kraft lignin (a & b) and low sulfonate alkali (c & d) lignin after dissolution at 160°C for 6 hrs with different biomass loading.

The non-polar lignin breakdown products extracted from the supernatant, along with their elution times during GC-MS, are shown in Table 2. The lignin breakdown products are observed to depend on the sample type and the dissolution temperature. For the technical lignins studied, guaiacol and allyl guaiacol were the major products at all biomass loadings (Figure 3). Higher quantities of guaiacol (5 g/kg of biomass) were produced from kraft lignin when compared to low sulfonate alkali lignin. The quantity of guaiacol produced (per kg of starting material) decreases as a function of increasing biomass loading. Higher quantities of ally guaiacol are produced from low sulfonate alkali lignin (2 g/kg) than from kraft lignin (1 g/kg). Other products like methyl guaiacol, ethyl guaiacol, vinyl guaiacol, vanillin, guaiacyl acetone are also present at smaller concentrations. Production of these minor products is observed to increase with increases in biomass loading from 3 wt% to 10 wt%, but is observed to decrease on further increasing the biomass loading to 20 wt%. Similar quantities of guaiacyl acetone are produced on dissolution of both kraft lignin and low sulfonate alkali lignin. Higher quantities of ethyl guaiacol, vinyl guaiacol and vanillin were produced from kraft lignin, whereas a higher quantity of methyl guaicol was obtained from low sulfonate alkali lignin. As these technical lignins were derived from softwood [29,30] and contain very small quantities of S-lignin in the original feedstocks, syringyl compounds were not significant. Similar compounds were observed by Stark et al. from the oxidative depolymerization of beech lignin [27] and by Reichert on electrolysis oxidative cleavage of alkali lignin [25].

**Table 2:** Non-polar lignin breakdown products found in the benzene extract of the supernatant

	**Name**	**Compound**	RT*
1	Guaiacol		8.8
2	4-Ethyl Guaiacol		10.4
3	4-Vinyl Guaiacol		11.1
4	Eugenol		11.61
5	Syringol		12.03
6	4-(1-propenyl) Guaiacol		12.97
7	Vanillin		13.54
8	Allyl Syringol		14.49
9	Guaiacylacetone		14.14
10 ^#^	Anthracene-d_10_		16.97

*Retention time (signifies retention time of the compounds in the GC/MS).

^#^ Internal standard.

The acidic or basic dissolution conditions that may be involved during the dissolution of lignin in IL can be predicted based on the products formed after dissolution. Vanillin has been previously produced from alkaline treatment and nitrobenzene oxidation of kraft lignin [31]. Adler et al. show the formation of guaiacol as a result of acidolysis of guaiacylglycerol-β-guaiacyl ether [32]. They show that the guaiacol is the main product due to the cleavage of β-aryl ether linkages. Adler et al. (1966) also predict guaiacylacetone to be one of the products of lignin acidolysis, but this monomer was present in small quantities in our supernatant solutions. Formation of aldehyde derivatives (coniferaldehyde) like allyl guaiacol during acid pretreatment of lignin is well cited in the literature [31-33]. Presence of higher quantities of guaiacol and allyl guaiacol indicated acidic dissolution conditions in IL under these conditions. We have recently published on the dual acidic and basic behavior of [C_2_mim][OAc] as a function of temperature, and the products identified here strongly confirm these previous findings [34].

### Lignin breakdown products from switchgrass

**Figure 4:**
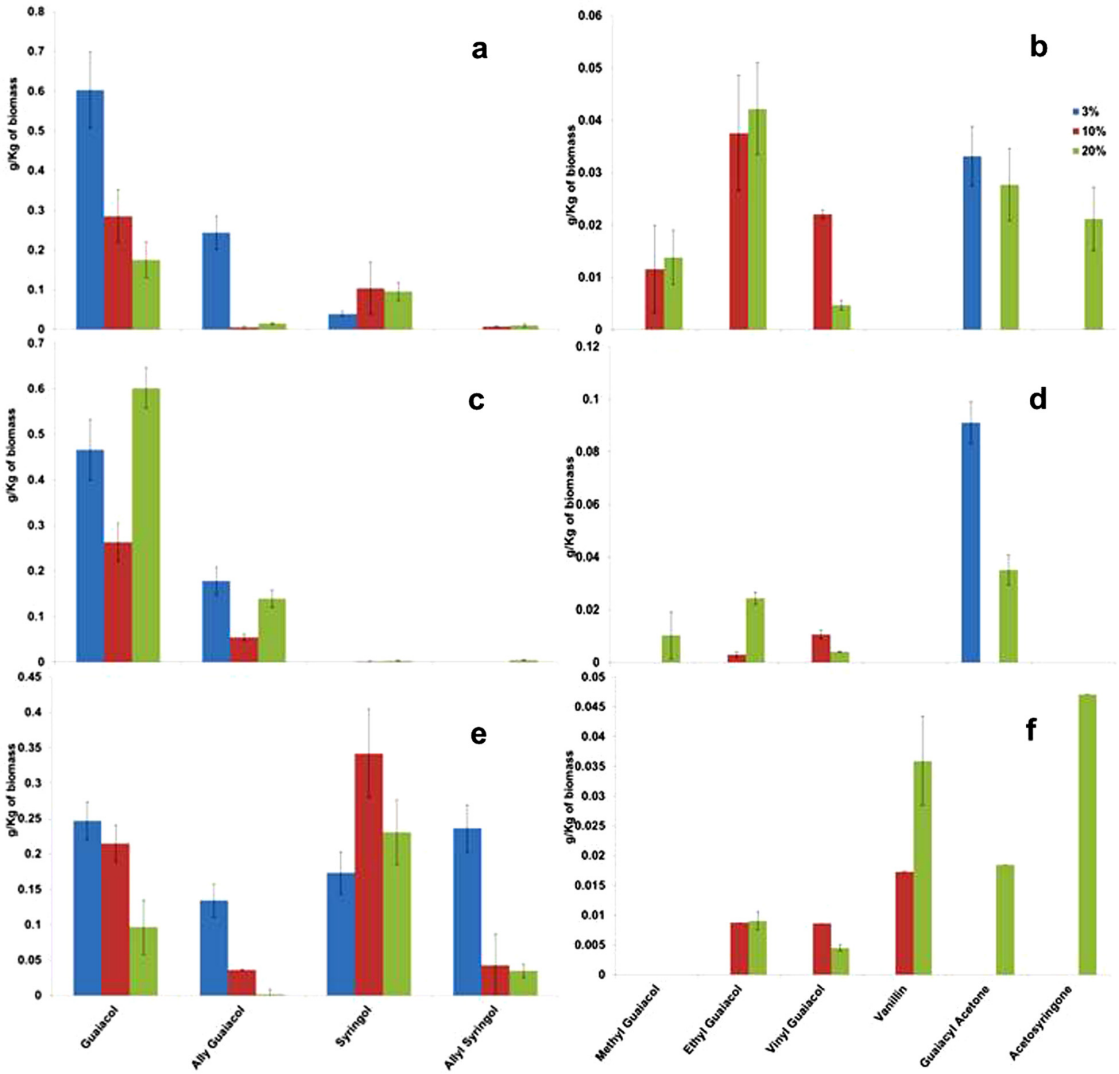
Major and minor lignin breakdown products from switchgrass (a & b), pine (c & d) and eucalyptus (e & f) after dissolution at 160°C for 6 hrs with different biomass loading.

The total amount of byproducts produced from lignocellulosic biomass was observed to be lower than that produced from technical lignins. This is reasonable given that lignin constitutes only ~1/3 of the dry weight of biomass. Guaiacol was obtained on dissolution of all lignocellulosic biomass in [C_2_mim][OAc] at 160°C for 6 hrs (Figure 4). Allyl guaiacol and syringol are also produced by dissolution of switchgrass (Figure 4a). As in the case of kraft lignin, the amount of guaiacol and allyl guaiacol produced decrease with increase in biomass loading. The production of syringol-type lignin compounds like syringol, allyl syringol and aceto syringone increases with increase in biomass loading. This increase implies breakdown of syringyl lignin increases with increase in biomass loading. All the minor products (methyl guaiacol, ethyl guaiacol, vinyl guaiacol, guaiacyl acetone and acetosyringone) except vanillin are produced on dissolution of switchgrass (Figure 4b), and the amount of these products increases with biomass loading. The increase in production of these minor compounds and decrease in production of major compounds indicates incomplete breakdown of lignin with increase in biomass loading.

### Lignin breakdown products from pine

Guaiacol and allyl guaiacol were the only major products from dissolution of pine (Figure 4c). This is expected as pine consists of mostly guaiacyl lignin and has low (or no) syringyl lignin. In the case of pine, no correlation between the biomass loading and the total amount of guaiacol released was observed. The quantity of guaiacol and allyl guaiacol produced decreases on increasing the biomass loading from 3% to 10% and further increases on increasing the biomass loading to 20%. Methyl guaiacol, ethyl guaiacol, vinyl guaiacol and guaiacyl acetone are produced as minor products from pine (Figure 4d). The amount of guaiacyl acetone decreases with increase in biomass loading. At higher biomass loadings there is a small increase in the minor products generated.

### Lignin breakdown products from eucalyptus

Unlike pine, eucalyptus contains a higher amount of syringyl lignin than guaiacyl lignin and all major products (guaiacol, allyl guaiacol, syringol and allyl syringol) are produced (Figure 4e). The amount of guaiacol and allyl guaiacol decreases with increases in biomass loading. The decrease in the production of these compounds indicated decrease in guaiacyl-lignin breakdown with increase in biomass loading. But the quantity of syringol produced increases on increasing the biomass loading from 3% to 10% and decreases on further increasing the biomass loading. Similar to dissolution of switchgrass, breakdown of guaiacyl-lignin decreases and syringyl-lignin increases with increase in biomass loading. At higher biomass loading levels of eucalyptus, increasing quantities of ethyl guaiacol, vanillin, guaiacyl acetone and acetosyringone were produced (Figure 4f).

As shown in the case of kraft lignin, changing the dissolution temperature changes the products that can be recovered from lignocellulosic biomass. A higher quantity of unsaturated guaiacols and aldehydes can be produced by decreasing dissolution temperature, and although not tested here, reaction time. It has been previously reported that vinyl guaiacol is produced from switchgrass when it is pretreated under alkaline conditions [32]. It has also been reported in the literature that guaiacylacetone is produced under acidic treatment conditions [32]. Guaiacol and syringol have been reported as the lignin products under acidic pretreatment conditions [32, 33]. The reaction mechanisms involved in the formation of these compound needs to be further investigated.

### Temperature dependence of lignin breakdown products

**Figure 5:**
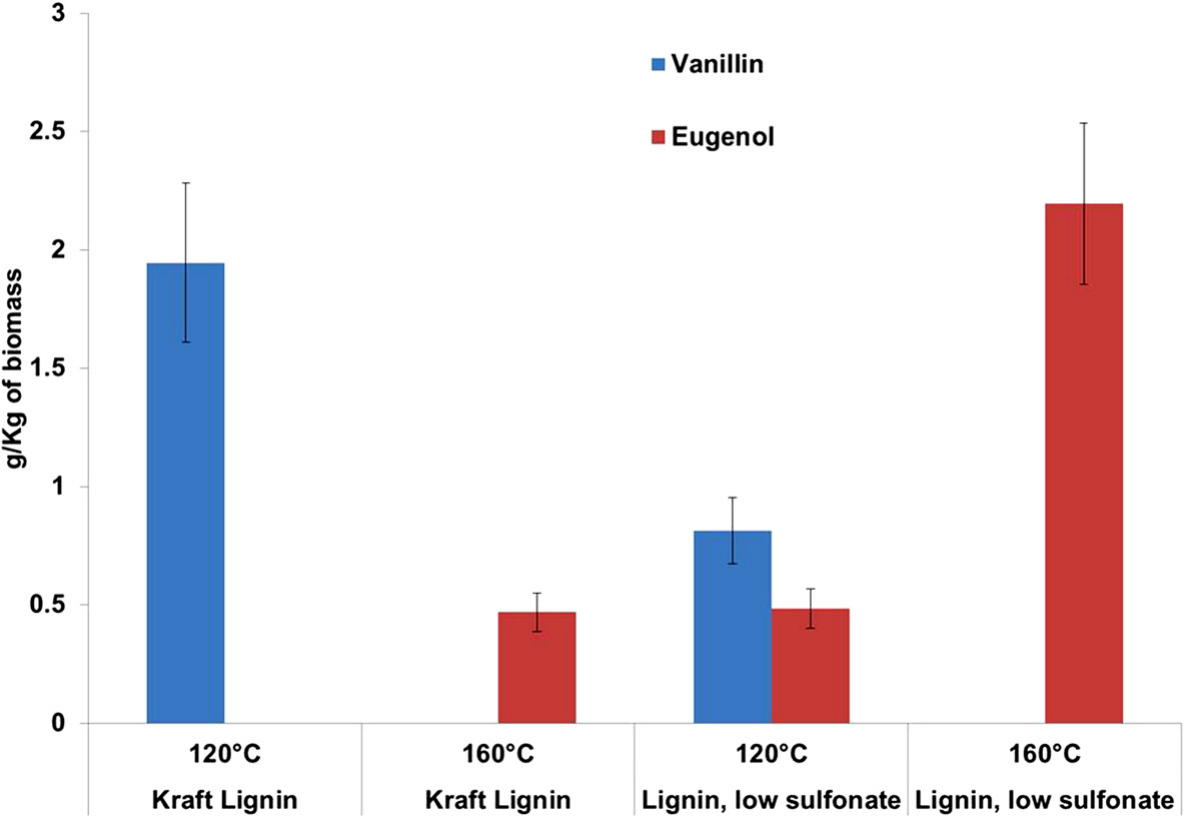
Lignin breakdown products (vanillin and eugenol) from low sulfonate alkali lignin and kraft lignin after dissolution at 120 and 160°C for 6 hrs at 3% biomass loading.

It is clear from the above discussions that the patterns of degradation products are very different from different biomass and also at different processing temperatures. Based on the final desired products, the dissolution conditions can be tuned to optimize the recovery of certain products. For example, the cleavage of the methyl ketone group from vanillin at higher temperature leads to guaiacol formation, and therefore if it is desired to generate more vanillin from biomass it can be obtained in higher quantities by lowering the process temperatures to 120°C (Figure 5). Similarly eugenol and vinyl guaiacol show the most dramatic impact of dissolution temperatures at the two temperatures studied. These examples provide evidence of a very flexible IL technology for lignin breakdown and product optimization. It is important to note that since the putative lignin glass transition temperature (polymeric lignin softens at broad temperature range instead of having a sharp melting point) is around 140-165°C based on the source of lignin [35], it is expected that polymeric lignin will be increasingly depolymerized at higher temperatures. The unique characteristics of [C_2_mim][OAc] induced behavior makes it a promising technology for the selective production of these chemicals while also serving as an efficient means of pretreating biomass [34].

## Conclusion

In this study [C_2_mim][OAc] was used to produce monomeric aromatic compounds from two types of technical lignins and three types of lignocellulosic biomass (pine, switchgrass, and eucalyptus) during pretreatment. Several guiacyl monomers were found to be present in the supernatant of technical lignins and biomass samples after dissolution in [C_2_mim][OAc] at 160°C for 6 hrs. Guaiacol was the common product from both technical lignins and biomass, and was produced at higher levels at 3% biomass loading. Higher biomass loadings did not generate more products per kg of starting material. Syringyl monomers were produced on dissolution of switchgrass and eucalyptus. Furthermore, the dissolution conditions can be changed to produce higher amount of the desired byproduct. The total amount of non-polar lignin products ranged from 0.5–5.7 g/kg of biomass. The amount of vanillin produced ranged from 0.04–2.0 g/kg of biomass under two processing temperatures tested. The products reported in this work only represents the non-polar monomeric components that were soluble in benzene after dissolution. Polar lignin products are expected to be present in the supernatant and efforts are underway to extract and quantify them. These results indicate that certain ILs used for pretreatment may also hold significant promise in the conversion of polymeric lignin to smaller aromatics and desired renewable chemical outputs.

## Materials and methods

Kraft lignin was supplied from MeadWestvaco Corp., Richmond,VA. Low sulfonate alkali lignin was purchased from Sigma Aldrich. Switchgrass (*Panicum virgatum*, cultivar MPV2) was provided by the laboratory of Dr. Ken Vogel. Samples of *Pinus radiata* and *Eucalyptus globulus* were provided by Arborgen. 1-ethyl-3-methylimidazolium acetate ([C_2_mim][OAc], 98% purity) was used as the solvent to depolymerize and dissolve lignin from the biomass. Benzene was used as the extraction solvent. Guaiacol, ethyl guaiacol, vinyl guaiacol, vanillin, eugenol, syringol, 4-allyl syringol, guaiacyl acetone and 2-methoxy, 4-propenyl phenol were used as standards to confirm the position and the mass to charge ratio of the Gas Chromatography/Mass Spectrometry (GC/MS) peaks. All other chemicals used in this study were purchased from Sigma Aldrich and used as received.

### Lignin dissolution in IL

Samples were ground to 40 mesh before the dissolution process (Thomas-Wiley Mini Mill fitted with a 40-mesh screen; Model 3383-L10 Arthur H. Thomas Co., Philadelphia, PA, USA). Technical lignins and biomass were dissolved in [C_2_mim][OAc] at 120 and 160°C in a conventional oven (Thelco Laboratory Oven, Jouan Inc, Virginia) for 6 hrs. Solid loading was varied from 3 wt% (300 mg in 9.7 mL of [C_2_mim][OAc]) to 10 wt% and 20 wt%, and components were mixed at room temperature before being placed in the oven. To this mixture 10 μL of anthracene-D_10_ was added as internal standard (IS), for quantification in GC/MS analysis. 35 mL of hot water (95°C) was added to the sample to precipitate the dissolved biomass (mostly glucans, unsolubilized lignin). The mixture of [C_2_mim][OAc], water, and biomass was then centrifuged to separate the solid (recovered biomass) and liquid ([C_2_mim][OAc] and water). This mixture of [C_2_mim][OAc] and water will be referred to as the supernatant for the rest of this report. After the collection of supernatant, the biomass was further washed ten times with 100 ml of water (10 ml/wash).

### Lignin extraction from the supernatant

A total of 10 mL of benzene was added to the supernatant in two steps. The resultant mixture (benzene+ sample) was mixed thoroughly and was then phase separated using a centrifuge. This allowed for all the non-polar compounds to be extracted from the supernatant to the benzene phase. This benzene solution was further concentrated under nitrogen to a final volume of 1 ml.

### Gas chromatography–mass spectrometry (GC-MS)

The analysis of the lignin breakdown compounds present after benzene recovery was performed using a GC-MS (Thermo Electron Corporation with Trace GC Ultra, Polaris-Q MS and TriPlus auto sampler). The compounds were separated using a TR-SMS (30 m, 0.25 mm ID, 0.25 μm) chromatographic column. 10 μl of the sample was injected into the GC at an inlet temperature of 220°C and was operated in a split mode (split flow of 12 mL/min, split ratio = 10). Helium was used as a carrier gas with a constant flow rate of 1.2 mL/min. The temperature of the GC was held at 45°C for 1 min, was then increased at a rate 10°C/min up to 300°C and was held at this temperature for 1 min. The MS was used until the end of GC run with a solvent delay of 3.5 min. The ion source was maintained at a temperature of 250°C and the MS was operated in scan mode. Anthracene-d10 was used as the internal standard as it is not present in the biomass samples. The standards of each compound were used to calculate the individual response factor.

### Quantification: calculation of product yield

Single-point calibration was used to calculate the response factor of the eluted compounds with respect to the internal standard (IS). Standards containing 2 mM of the compound and 20 μg of IS in 1 ml of Benzene were used for calibration. The area under the spectral peak of the compound and the IS were used to calculate the response factor (RF) (equation 1).

(1) $$\text{RF}=\frac{\text{Are}a_{\text{compound}}/\text{weight}\;\text{of}\;\text{the}\;\text{compound}\;\text{injected}}{\text{Are}a_{\text{IS}}/\text{weight}\;\text{of}\;\text{IS}\;\text{injected}}$$

The response factor was then used to calculate the actual concentration of the compounds in the sample. Area under the spectral peak of the compound and the IS for an actual sample are used to calculate the concentration of the compound in the injected volume of the sample.

(2) $$\begin{array}{c} \text{Weight of the compound in the injected sample}\\ \;=\frac{\text{Are}a_{\text{compound}}}{\text{Are}a_{\text{IS}}}\times \frac{\text{weight}\;\text{of}\;\text{IS}}{\text{RF}} \end{array}$$

RFs were determined independently for each standard using GC/Ms.

The amount of each product was calculated as

(3) $$\frac{\text{Amount}\;\text{of}\;\text{product}\;A\;\text{in}\;\text{the}\;\text{supernatant}\left(g\right)}{\text{Amount}\;\text{of}\;\text{the}\;\text{starting}\;\text{biomass}\;\left(\text{Kg}\right)}$$

## Competing interests

The strategy described in this paper has been included in a patent application.

## Authors’ contributions

SS designed and coordinated the study; PV, PS, MA, PDA, BAS and SS conducted the experiments and data analysis. PV, BAS and SS wrote the manuscript and all authors read and approved the final manuscript.
